# Toxicity and Sublethal Effects of Lambda-Cyhalothrin Insecticide on Parent and Filial Generations of *Henosepilachna vigintioctomaculata* (Coleoptera: Coccinellidae)

**DOI:** 10.3390/insects16030259

**Published:** 2025-03-03

**Authors:** Wenbo Li, Muhammad Naeem, Juan Cui, Guochuan Du, Huanhuan Chen

**Affiliations:** 1 College of Biological Resource and Food Engineering, Qujing Normal University, Qujing 655011, China; lwbnicklwb@163.com (W.L.); naeem1633@yahoo.com (M.N.); 2 College of Agriculture, Jilin Agriculture Science and Technology College, Jilin 132101, China; cuijuanjilin@163.com; 3 College of Geography and Tourism, Qujing Normal University, Qujing 655011, China

**Keywords:** *Henosepilachna vigintioctomaculata*, lambda-cyhalothrin, life table, transgenerational studies, detoxifying enzyme

## Abstract

Lambda-cyhalothrin is a synthetic pyrethroid insecticide that is widely used to control leaf-eating pests. Because of increased insecticide resistance, an understanding of sublethal cross-generational effects of insecticides is important. We examine the effects of sublethal concentrations (SLCs) (LC_10_, LC_20_, and LC_40_) of lambda-cyhalothrin on the growth, reproduction, and detoxification enzyme activities of F_0_ and F_1_ generation *Henosepilachna vigintioctomaculata*. Lambda-cyhalothrin is toxic to adult *H. vigintioctomaculata*, with an LC_40_ at 48 h of 0.355 mg L^−1^. At SLCs, lambda-cyhalothrin significantly reduces the longevity and average fecundity of F_0_ and F_1_ adults, and prolongs the durations of the egg, larval, and pupal stages and adult preoviposition period. Additionally, an increased lambda-cyhalothrin concentration significantly decreases net reproductive rates, and both finite and intrinsic rates of increase in the F_1_ generation, and significantly increases the average generation cycle. The detoxification enzyme activity of F_1_ adults treated with SLCs of lambda-cyhalothrin for 48 h trends upwards. Results indicate that low concentrations of lambda-cyhalothrin induce glutathione S-transferase and carboxylesterase activities and inhibit multifunctional oxidase activity. The growth, development, and reproduction of the *H. viltioctomaculata* F_1_ population remain inhibited by lambda-cyhalothrin treatment in the adult stage, and inhibitory effects increase with increased lambda-cyhalothrin concentration. The control efficacy of lambda-cyhalothrin against *H. viltioctomaculata* shows cross-generational effects.

## Simple Summary

The ladybug *Henosepilachna vigintioctomaculata* is a widely distributed leaf-eating pest. Lambda-cyhalothrin, a synthetic pyrethroid insecticide, is widely used to control leaf-eating pests. However, the effects of this insecticide on biological activity, cross-generations, and detoxification enzyme activity of this ladybug are imperfectly known. We reared *H. vigintioctomaculata* for at least three generations under experimental conditions to determine the effects of lambda-cyhalothrin on biological activity. We evaluated the sublethal effects on F_0_ generation adults, and cross-generational effects on F_1_ adults, using age–stage bisexual life tables, and examined the detoxification enzyme activity of F_0_ adults. Sublethal concentrations of lambda-cyhalothrin significantly reduced F_0_ adult longevity and average fecundity, and inhibited various life table parameters in the F_1_ population; there was also a cross-generational genetic effect, with population growth being inhibited. Low concentrations of lambda-cyhalothrin significantly inhibit *H. vigintioctomaculata* population growth. Multifunctional oxidase, carboxylesterase, and glutathione S-transferase play important roles in *H. vigintioctomaculata* resistance to lambda-cyhalothrin.

## 1. Introduction

The 28-spotted ladybug *Henosepilachna vigintioctomaculata* (Coleoptera: Coccinellidae) is a crop pest that occurs widely throughout China, and elsewhere in countries such as Korea, Japan, Russia, and Australia. This ladybug is a phytophagous pest that feeds mainly on crops such as Solanaceae, Leguminosae, Cruciferae, and Cucurbitaceae, with adults and larvae eating the tender leaves and stems [1,2]. When leaves are eaten, a semi-transparent parallel-shaped depression is formed, and in severe cases, only leaf veins and the epidermis remain, causing the leaves to wilt, yellow, and die. China is an important producer of potato—a globally important food crop—and *H. vigintioctomaculata* seriously affects potato agriculture [2,3,4]. Changes in microclimates and promotion of intercropping of corn, vegetables, and potatoes have also led to *H. vigintioctomaculata* becoming both an increasingly serious pest in potato plantations in the Yunnan, Guizhou, and Sichuan regions, and a main spreader of potato brown spot disease, which also poses a threat to local potato production. In severe cases, yields can be reduced by 50% [5,6,7].

The insecticide lambda-cyhalothrin is highly efficient at controlling leaf-eating pests [8]. After spraying, insecticide residue, in general, gradually decreases over time, and with crop growth [9]. The physiology and behavior of some pests exposed to insecticides at sublethal doses may change [10,11]. Most sublethal insecticide doses have inhibitory or delayed effects on pest populations, but some can also stimulate growth and development, and promote pest proliferation [12]. These sublethal effects on pests can lead to physiological and biochemical changes and insecticide resistance. Therefore, understanding the sublethal effects of insecticides on pests is important to evaluate their efficacy and assess the risks associated with their use [13]. Pests can reduce the effects of insecticides by regulating detoxification enzyme activities [14]. Among enzymes, glutathione S-transferase (GST), carboxylesterase (CarE), and multifunctional oxidase (MFO) are the most important. Low concentrations of insecticides or plant secondary metabolites can induce or inhibit the activity of various enzymes, thereby affecting pest metabolic processes, promoting resistance and providing continuous selection pressure, reducing the effectiveness of insecticides for pest control [15,16].

While the median lethal concentration (LC_50_) or median lethal dose (LD_50_) have been commonly used to evaluate the effects of insecticides on pests, they only report the response to insecticides at certain developmental stages [17]. Life tables can be used to more comprehensively analyze the effects of pesticides on pests at the population level. Compared with traditional life tables, an age–age bisexual life table increases statistics for males, accurately describes age differentiation of insects, differentiates between the pre- and total-oviposition periods of adults, and can comprehensively describe changes in the entire population [18,19,20]. Beta-cypermethrin, phoxim, and abamectin pesticides are mostly used to control potato ladybird adults [4].While the sublethal effects of insecticides on target species are being increasingly explored, the effects of the lethal concentration (LC) of lambda-cyhalothrin on the growth, development, and fecundity of *H. vigintioctomaculata* are imperfectly known. We first determine the sublethal concentration (SLC) of lambda-cyhalothrin on the F_0_ generation, and its effect on the F_1_ generation at SLCs. We construct an age–instar bisexual life table for the F_1_ population and compare changes in parameters such as developmental duration, survival rate, fecundity, and longevity at different lambda-cyhalothrin concentrations. We also report the detoxifying enzyme activities after SLC treatment of F_0_ adults. The results provide a theoretical basis for a comprehensive assessment of the potential of lambda-cyhalothrin to control *H. vigintioctomaculata*, and for more informed applications of this insecticide.

## 2. Materials and Methods

### 2.1. Insect Rearing

*Henosepilachna vigintioctomaculata* were collected from Luliang County, Qujing City potato fields, Yunnan Province (103°48′52.02″ E, 25°17′20.74″ N, altitude 1878 m) in September 2023. Insects were placed in an insect feeding cage (50 × 50 × 50 cm) at room temperature. Potted potatoes (Yunshu 108) grown at (25 ± 1) °C, relative humidity 70% ± 5%, and a photoperiod 16:8 h L/D were cultivated as a host; fresh leaves were cut to feed the ladybugs when the plants had grown to 15–20 cm. Before experimentation, adult ladybugs were reared for at least three generations with no exposure to insecticide. Three-day-old adults were used as the initial insect source. Insect feeding conditions were consistent with potato cultivation conditions.

### 2.2. Insecticide and Chemicals and Enzyme Assay Kits

The stock insecticide solution was 2.5% lambda-cyhalothrin WG (Henan Yongguan Qiaodi Agricultural Science and Technology Co., Ltd, (Zhengzhou, China). Multifunctional oxidase (MFO) activity detection kits (A162-1-1), Carboxylesterase (CarE) activity detection kits (A133-1-1), and Glutathione S-transferase (GSH-ST) activity detection kits (A004-1-1) were purchased from Nanjing Jiancheng Bioengineering Institute Co., Ltd (Nanjing, China).

### 2.3. Determining Biological Activity

The sensitivity of *H. vigintioctomaculata* to lambda-cyhalothrin was determined using a leaf film method [21]. Five lambda-cyhalothrin treatments (1.25, 1.00, 0.75, 0.50, and 0.25 mg L^−1^) were initially established; water was used as a control. Each treatment contained 30 × 3 d old similarly sized adult ladybugs, and was replicated three times. Before experimentation, adult ladybugs were starved for 48 h. Potato leaves were immersed in each treatment concentration for 20 s, then removed, placed in an insect box (15 × 12 × 5 cm), dried, and their petiole wrapped with water-soaked cotton wool. Ladybugs were then added. Ladybug mortality was recorded after 48 h of treatment. Feeding conditions were the same as in Section 2.1. Ladybugs were pronounced dead if touched by a brush and no reaction occurred. A virulence regression curve, 95% confidence intervals, chi-square values (χ^2^), and degrees of freedom (*df*) were calculated for LC_10_, LC_20_, and LC_40_ concentrations.

### 2.4. Effects of Lambda-Cyhalothrin Sublethal Treatment on F_0_ and F_1_ H. vigintioctomaculata

Potato leaves were immersed in LC_10_, LC_20_, and LC_40_ concentrations for 20 s, then removed and dried; water was used as a control. Sixty healthy 3 d old similarly sized male and female adults (1:1, morphological identification) were fed potato leaves and reared in an artificial climate box [4]. After 48 h of exposure to leaves immersed in different concentrations of insecticide (without changing leaves), surviving *H. vigintioctomaculata* were removed, and males and females were paired, placed in a Petri dish, and fed fresh, untreated potato leaves. The initial stage of the F_0_ generation experiment involved approximately 30 individuals, with three biological replicates. At least 15 pairs of ladybugs from each insecticide treatment were paired; the fresh potato leaves in the insect box were replaced daily. The oviposition date, oviposition amount, and adult longevity of single females were observed regularly (8:00 and 18:00 h). If a male died before a female, it was replaced. We refer to adult contemporary *H. vigintioctomaculata* treated with SLCs of lambda-cyhalothrin as the parent generation (F_0_), and offspring produced by natural mating of these parents as the filial generation (F_1_) [2].

Eggs (90, oviposition < 5 h) laid on the same day by an F_0_ female in each lambda-cyhalothrin treatment and the control group were used as the F_1_ generation. These eggs were placed into circular plastic, numbered Petri dishes (diameter 60 mm) with moist filter paper, and then moved to an artificial climate box for hatching, where the hatching rate was determined. The feeding conditions were the same as in Section 2.1. Individual newly hatched F_1_-generation larvae treated with different insecticide concentrations were inoculated separately into Petri dishes for feeding. Fresh potato leaves were placed into Petri dishes, and ladybug development was monitored every 24 h. After 24 h of pupation, pupae were weighed, then transferred into individual centrifuge tubes sealed with absorbent cotton, and returned to the climate chamber. After adult eclosion, pupal duration was determined. For each insecticide concentration treatment, female and male adults that emerged on the same day were randomly selected and paired 1:1 and placed into new culture dishes. A wet cotton ball was placed in the dish to provide moisture, and fresh leaves were replaced the next day. Test insects ate and deposited eggs. The number of eggs laid by a single female and the longevity of adult females and males were observed daily (8:00 and 18:00 h) until all adults had died. The number of eggs hatched/the total number of eggs laid by females represents the egg-hatching rate. The time from newly hatched larvae to pupation represents the larval development period, and the time from pupation to adult eclosion represents the pupal development period. The percentage of the number of pupae that could break the shell and become adults divided by the total number of pupae × 100 represents the eclosion rate.

### 2.5. Determining Detoxifying Enzyme Activities

Detoxifying enzyme activities were determined using the method of Jiang et al. [22]. F_0_-generation adult ladybugs were fed fresh leaves treated with LC_10_, LC_20_, and LC_40_ concentrations of lambda-cyhalothrin, with four replicates per treatment. After 48 h, surviving adults were collected and placed into centrifuge tubes. Each replicate contained 10 individuals, frozen in liquid nitrogen and stored at −80 °C for enzyme activity determination. Ten similarly sized adults were selected from each concentration treatment. PBS (pH 7.4) was added to an ice bath homogenate at a weight (g)–volume (mL) ratio of 1:9; the homogenate was centrifuged at 4 °C and 12,000 r/min for 30 min. The supernatant was recovered to determine GST, CarE, and MFO enzyme activities, and protein concentration, following kit instructions.

### 2.6. Data Analysis

A probability unit analysis was used to analyze the toxicity regression equation of lambda-cyhalothrin using SPSS 26.0 (IBM Co., Ltd., Armonk, NY, USA); SLCs were determined. One way ANOVA was used on data related to detoxification enzymes. Tukey’s multiple range test was used for multiple comparisons, and Student’s t tests were used to identify significant differences between treatments in pairwise comparisons. Original growth and development, survival rate, and fecundity data for the F_1_ generation were collated and imported into Twosex-MSChart software (v 5/7/2024) to calculate developmental duration, adult longevity, adult preoviposition period (APOP), total preoviposition period (TPOP), fecundity, net reproductive rate (*R*_0_), finite rate of increase (λ), intrinsic rate of increase (*r*), and average generation cycle (*T*) for each stage [23,24,25,26]. All variances and standard errors were obtained by using 100,000 random sampling bootstraps, and the differences for each parameter of the insecticide treatments were evaluated by paired bootstrap tests [27,28]. Plots were generated using SigmaPlot 14.0. The specific calculation formulas are as follows:

(1) $$l_{x}=\sum_{j=1}^{\beta }s_{xj}$$

(2) $$M_{x}=\frac{\sum_{j=1}^{\beta }S_{xj}f_{xj}}{\sum_{j=1}^{\beta }S_{xj}}$$

(3) $$R_{0}=\sum_{x=0}^{\infty }l_{x}m_{x}$$

(4) $$\sum_{x=0}^{\infty }e^{-r(x+1)}l_{x}m_{x}=1$$

*λ* = *e^r^* (5)

(6) $$T=\frac{\ln(R_{0})}{r}$$

## 3. Results

### 3.1. Toxicity of Lambda-Cyhalothrin to Henosepilachna vigintioctomaculata Adults

After 48 h treatment, the LC_10_, LC_20_, and LC_40_ values for lambda-cyhalothrin exposure to *H. vigintioctomaculata* adults were 0.193, 0.251, and 0.355 mg L^−1^, respectively. Three concentration values overlapped with other 95% CL values for subsequent experiments (Table 1).

### 3.2. Effect of Sublethal Concentration of Lambda-Cyhalothrin on Longevity and Fecundity of F_0_ H. vigintioctomaculata

LC_10_, LC_20_, and LC_40_ concentrations of lambda-cyhalothrin significantly affected the longevity and average fecundity of F_0_ adults (Table 2). Compared with the control group, the higher the concentration of lambda-cyhalothrin, the shorter the adult life span (female and male), with the life span of females being slightly longer. After exposure to lambda-cyhalothrin concentrations (LC_10_, LC_20_, and LC_40_), individual egg production and F_1_ egg hatching rates also significantly decreased compared with the control group.

### 3.3. Effect of Sublethal Concentration of Lambda-Cyhalothrin on Growth, Development, Fecundity, and Pupal Weight of F_1_ H. vigintioctomaculata

**Figure 1:**
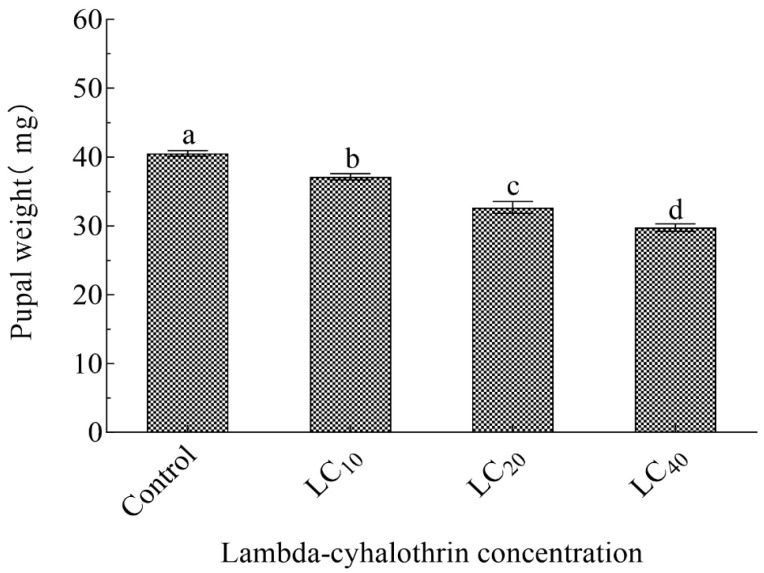
Pupal weight of progeny of lambda-cyhalothrin-treated *H. vigintioctomaculata*. Note: Data are means ± SE; different lowercase letters above whiskers indicate significant differences (*p* < 0.05, Tukey’s multiple range test).

LC_10_, LC_20_, and LC_40_ concentrations of lambda-cyhalothrin affected each F_1_ generation developmental stage (Table 3). Compared with the control, the duration of the egg, larval, and pupal stages in the LC_10_ and LC_20_ treatment groups differed significantly (*p* < 0.05); the lifespan of male and female adults in the LC_40_ concentration treatment group was 16.57% and 19.76%, significantly shorter than control group adults. APOP and TPOP were prolonged with increased lambda-cyhalothrin concentrations; the control group’s APOP and TPOP durations were the shortest (6.20 d and 23.53 d), and those in the LC_40_ treatment were the longest (7.10 d and 30.45 d, respectively). Compared with other treatments, the numbers of eggs laid by females exposed to the LC_40_ concentration was lowest (13.40 eggs/female); the number of eggs laid by females in the control group (55.59 eggs/female) was 1.13×, 1.70×, and 4.15× that of the LC_10_, LC_20_, and LC_40_ treatments, respectively. Additionally, compared with controls, the pupal weight and adult emergence rate of the F_1_ generation were also significantly lower in the LC_10_, LC_20_, and LC_40_ treatments (*p* < 0.05), with the effect stronger with increased insecticide concentration (Figure 1, Table 3).

### 3.4. Population Parameters

Compared with the controls, the LC_10_, LC_20_, and LC_40_ treatments significantly affected the F_1_ generation’s life table parameters. The values for the *r*, λ, and *R*_0_ of the F_1_ population decreased with increased lambda-cyhalothrin concentration, and *T* increased (Table 4).

### 3.5. Age–Stage Specific Maternity

**Figure 2:**
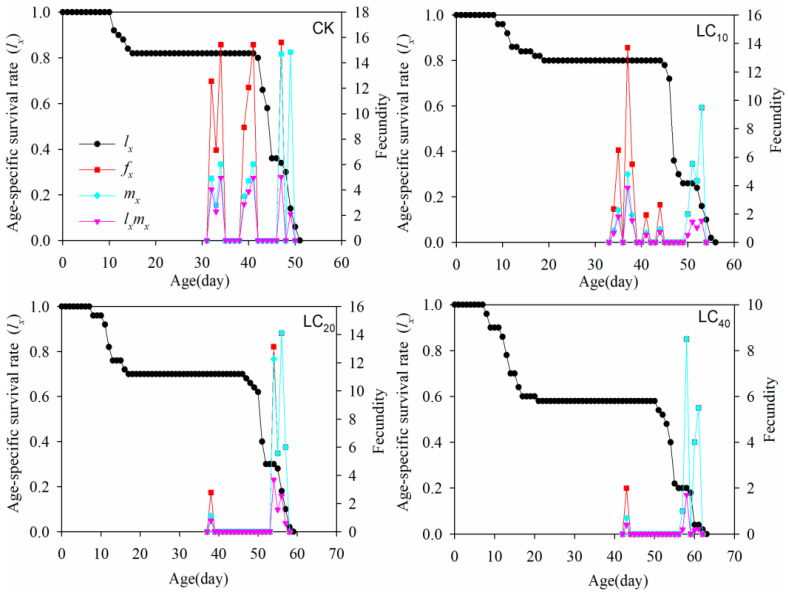
*l_x_*, *f_x_*, *m_x_*, and *l_x_m_x_* values of F_1_ *H. vigintioctomaculata* descended from F_0_ ladybugs treated with lambda-cyhalothrin and control values.

At each concentration, the *l_x_* curves follow a similar trend: there is a period of stability, and then a gradual and then steep decline (Figure 2), indicating that the death of individuals in the *H. vigintioctomaculata* F_1_ population mainly occurred in the later stages. The *f_x_* curves are more variable, indicating greater variation in the emergence and oviposition of female adults, resulting in the curve appearing high and low. For the LC_10_, LC_20_, and LC_40_ lambda-cyhalothrin concentrations, peak adult female *f_x_* values were 13.77 (37 d), 14.11 (55 d), and 8.46 (58 d) (Figure 2). The peak number of eggs of *f_x_* in the control group was greatest (15.62 eggs d^−1^). Over time, the age-specific survival rate *l_x_* of the control group trended downwards. The *m_x_* curve indicated that the control group began reproducing at 33 d, whereas in other concentrations reproduction began 2–11 d later. Multiple peaks appear in the *mx* curve, indicating changes in individual spawning periods. The age-specific oviposition rate *l_x_m_x_* in the LC_40_-treated individuals decreased sharply, from 5.00 individuals d^−1^ in the control group to 1.70 individuals d^−1^ (Figure 2).

### 3.6. Effect of Lambda-Cyhalothrin Exposure on Detoxifying Enzyme Activity

**Figure 3:**
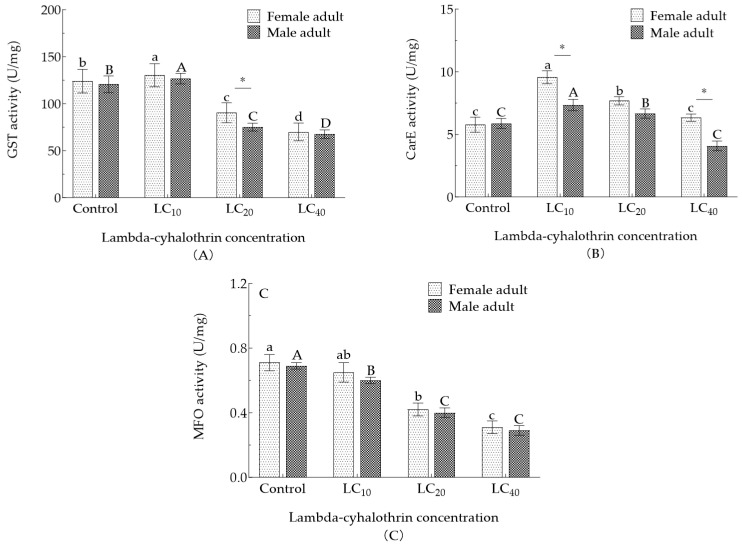
(**A**) GST, (**B**) CarE, and (**C**) MFO activities in *H. vigintioctomaculata* adults treated with lambda-cyhalothrin. Note: Data are means ±SE; different uppercase and lowercase letters indicate significant differences between males and females, respectively (*p* < 0.05, Tukey’s multiple range test). Asterisks indicate significant differences in detoxifying enzyme activities between females and males (* *p* < 0.05, independent samples *t*-test).

The detoxifying enzyme activities in the adult *H. vigintioctomaculata* treated with SLCs of lambda-cyhalothrin trended upwards (Figure 3). After 48 h exposure at LC_20_ and LC_40_ concentrations, female GST activities decreased significantly by 28.88% and 44.88% compared with control values, and male activities decreased significantly by 39.39% and 44.95%, respectively (*p* < 0.05); at LC_10_ there was an increase in GST activity. CarE activity first increased, then decreased with increased lambda-cyhalothrin concentration; the enzyme activities in the LC_40_ treatment were significantly lower than those in either of the other treatments (*p* < 0.05). With an increased treatment concentration, MFO activity was inhibited. Additionally, the GST activity of adults in the LC_20_ treatment and CarE activity in the LC_10_ and LC_40_ treatments differed significantly (*p* < 0.05).

## 4. Discussion

Insecticides are used extensively in agriculture, and they contribute to the development of pest resistance [17]. Integrated pest-management programs underscore the importance and necessity of moderate insecticide use [29]. Lambda-cyhalothrin is an efficient, broad-spectrum and quick-acting pyrethroid insecticide developed by ICI, UK. It has contact and stomach poisoning effects, and has no internal absorption effect. It is mainly used to control pests with chewing or piercing and sucking mouthparts [8,9,14]. Using a leaf-dip method, we evaluated the bioactivity of lambda-cyhalothrin against adult *H. vigintioctomaculata* [4]. Our LC_40_ value of 0.355 mg L^−1^ indicates that this insecticide exhibits potent toxicity toward *H. vigintioctomaculata*. Chemical control is the main strategy to manage *H. vigintioctomaculata*. However, post-application in the field, insecticide toxicity generally decreases because of various abiotic environmental factors, producing SLCs [30]. This reduced toxicity can induce sublethal effects in certain pest individuals that may be passed on to subsequent generations. Sublethal effects may influence the growth, development, behavior, and reproductive capacity of target pests and their offspring, and change population dynamics [30,31,32].

Many studies have reported that sublethal insecticide concentrations exert significant inhibitory effects. For instance, treatment with chlorantraniliprole at LC_10_ concentrations significantly reduces the survival rate and longevity of F_1_ generations of *Sogatella furcifera* [33]. Studies on the development of *Cydia pomonella* under lambda-cyhalothrin stress have yielded similar results [34]. We report SLCs of lambda-cyhalothrin (LC_10_, LC_20_, and LC_40_) to significantly reduce the lifespan and average oviposition of the F_0_ generation *H. vigintioctomaculata* compared with the control group ladybugs (Table 2). Furthermore, exposure to LC_40_ concentrations significantly reduced the F_1_ population size, with delays observed in egg, larval, and pupal developmental duration, further indicative of sublethal effects (Table 3). These findings are consistent with those for exposure of *Paracoccus marginatus* to SLCs of spirotetramat, with extended nymphal periods from the F_0_ to F_2_ generations and pre-adult delays [35]. We also report the pupal weight—an important indicator of insect stress resistance and environmental adaptability—of the F_1_ generation of *H. vigintioctomaculata* to decrease with increased SLC of lambda-cyhalothrin [36]. Leaves treated with LC_40_ concentrations also showed negligible feeding damage, likely attributable to the antifeedant properties of lambda-cyhalothrin [37]. Reduced feeding reduced the growth and development of *H. vigintioctomaculata*, and reduced body weight.

The impact of insecticides on pest fecundity manifests as sublethal effects [31]. The toxic excitatory response is influenced by various factors, with exposure duration (or generational exposure) being a key determinant [38]. Low doses of imidacloprid stimulate peach aphids, leading to increased methylation levels in the F_2_ generation compared with the F_1_ generation. This phenomenon may be linked to genetic adaptability induced by low-dose insecticide stress [39]. We report that SLCs of lambda-cyhalothrin also induce transgenerational effects in *H. vigintioctomaculata*. Specifically, the average oviposition, *r*, λ, and *R*_0_ values of both the F_0_ and F_1_ generations decreased with increased lambda-cyhalothrin concentration, limiting F_1_ population growth (Table 2 and Table 3). A similar phenomenon is reported for pests such as *Paracoccus marginatus*, *Aphis gossypii*, and *Rhizoglyphus robini* [9,14,40]. These findings suggest that insecticide treatments can effectively slow transgenerational population growth of a variety of species. This inhibitory effect may be attributed to the downregulation of gene expression associated with vitellogenin (Vg) and its receptor synthesis in females exposed to sublethal doses of insecticide. For example, the reduced expression of Vg and vitellogenin receptor (VgR) genes leads to decreases in their respective contents [40,41,42]. Furthermore, age–stage-specific maternity curves (Figure 2) indicate that lambda-cyhalothrin stress significantly suppresses the F_1_ generation’s reproductive capacity, with the effect increasing at higher concentrations. Interestingly, many insecticides (e.g., triazophos and thiamethoxam) regulate pest fecundity by modulating the expression of Vg and VgR through the TOR protein kinase and juvenile hormone signaling pathways [43,44]. Whether lambda-cyhalothrin similarly regulates the reproductive capacity of *H. vigintioctomaculata* adults via these pathways is unknown.

The reduced sensitivity of insects to insecticides typically involves multiple mechanisms, with the most prominent being the regulation of insecticide metabolism through detoxifying enzymes [15]. These enzymes play important roles in the metabolic processing of chemicals in insects. Insecticides can induce or inhibit their activity, and by exerting continuous selective pressure, drive the evolution of insecticide resistance [45]. Insecticides can affect various population parameters in insects such as developmental periods, reproductive capacity, and adult longevity. Additionally, they can influence the activity of detoxifying enzymes [46]. Different insecticides or plant secondary metabolites have distinct inhibitory or inducing effects on detoxifying enzyme activities in the same pest species [31]. For example, after 24 h of spirotetramat exposure, low concentrations induced a significant increase in GST and CarE activity in *Bradysia odoriphaga* [47]. Similarly, treatment with lambda-cyhalothrin significantly increased GST activity in *Cydia pomonella*, but suppressed CarE activity [48]. We report that, after 48 h of exposure to lambda-cyhalothrin, LC_10_ concentrations induced an increase in both GST and CarE activities in adult *H. vigintioctomaculata*. At LC_20_, the CarE activity in adult males and females was significantly higher than in the control group, but lower than in the LC_10_ treatment group, possibly because of the lower insecticide dose (where CarE was activated to participate in insecticide metabolism). Additionally, the GST activity of adults in the LC_20_ treatment and CarE activity in the LC_10_ and LC_40_ treatments differed significantly (*p* < 0.05). Similar GST and CarE activity results were reported by Kinareikina [49]. At increased dosage, enzyme activity was progressively suppressed. The degree of MFO activity correlated positively with insecticide dosage. At low lambda-cyhalothrin concentrations, the activities of CarE and GST in *H. vigintioctomaculata* were induced, but MFO activity was significantly reduced. Further molecular-level studies on higher resistance strains are required to elucidate the underlying mechanisms of resistance to lambda-cyhalothrin.

After the exposure of adult *H. vigintioctomaculata* in the F_0_ generation to SLCs of lambda-cyhalothrin, F_1_ population growth was significantly reduced, and the degree of reduction increased with increased insecticide concentration. This suggests that field application of lambda-cyhalothrin exerts transgenerational effects on *H. vigintioctomaculata*. In agricultural practice, the rational use of lambda-cyhalothrin to mitigate damage caused by *H. vigintioctomaculata* to crops is viable. However, the dosage and frequency of application must be managed to avoid enhancing pest fitness or promoting resistance through prolonged use of a single class of insecticide. Furthermore, the implementation of insecticide rotation—using two or more insecticides with different modes of action—could delay the development of resistance and support sustainable pest management strategies.

## 5. Conclusions

As a new insecticide with a high efficiency, low toxicity, and low residue, lambda-cyhalothrin has high insecticidal activity against Coleoptera pests. We report the effects of SLCs of this insecticide on the growth and development, longevity, and fecundity of *H. vigintioctomaculata* using an age–age bisexual life table. Compared with the control group, male and female longevity and fecundity all decreased, and other life table parameters deceased with increased SLCs. At low concentrations, this insecticide also increases the activity of detoxifying metabolic enzymes. Field application of lambda-cyhalothrin has a cross-generational effect on *H. vigintioctomaculata* that may affect F_1_ generations. This provides a reference for how to improve the evaluation of the effects of insecticides.

## Figures and Tables

**Table 1:** Sublethal concentrations of lambda-cyhalothrin for newly hatched adult *H. vigintioctomaculata* (F_0_) 48 h after treatment.

Insecticide	Number of Samples	LC_10_ (95% CL) (mg L^−1^)	LC_20_ (95% CL) (mg L^−1^)	LC_40_ (95% CL) (mg L^−1^)	χ^2^ (*df*)	Slope ± SE	*p*-Value
Lambda-cyhalothrin	450	0.193 (0.112–0.265)	0.251 (0.184–0.352)	0.355 (0.258–0.432)	9.53	3.91 ± 0.59	0.023

**Table 2:** Impact of sublethal concentrations of lambda-cyhalothrin on F_0_ generation adult *H. vigintioctomaculata* longevity and fecundity.

Parameter	Control	Lambda-Cyhalothrin (LC_10_)	Lambda-Cyhalothrin (LC_20_)	Lambda-Cyhalothrin (LC_40_)
	Mean ± SE	Mean ± SE	Mean ± SE	Mean ± SE
Male longevity (d)	24.39 ± 0.30 a	24.00 ± 0.23 a	21.45 ± 0.29 b	20.09 ± 0.33 c
Female longevity (d)	26.27 ± 0.34 a	26.00 ± 0.21 a	24.29 ± 0.39 b	21.79 ± 0.29 c
Fecundity (eggs laid/female)	65.66 ± 9.21 a	59.11 ± 7.09 b	42.12 ± 5.56 c	31.09 ± 4.76 d
F_1_ egg Hatching rate	84.29 ± 11.55 a	68.73 ± 9.79 b	58.26 ± 9.60 c	49.46 ± 7.88 d

Note: Different letters within a row indicate significant differences (*p* < 0.05).

**Table 3:** Fecundity and duration of various life-history parameters of progeny of lambda-cyhalothrin-treated *H. vigintioctomaculata*.

Parameter (Duration, Days)	Treatment			
	Control	Lambda-Cyhalothrin (LC_10_)	Lambda-Cyhalothrin (LC_20_)	Lambda-Cyhalothrin (LC_40_)
	Mean ± SE	Mean ± SE	Mean ± SE	Mean ± SE
Egg (d)	5.66 ± 0.07 c	6.48 ± 0.07 b	6.70 ± 0.07 b	7.12 ± 0.05 a
Larva (d)	15.05 ± 0.17 c	16.32 ± 0.23 b	16.60 ± 0.21 b	18.69 ± 0.30 a
Pupa (d)	4.59 ± 0.08 c	5.40 ± 0.13 b	5.71 ±0.15 b	6.17 ± 0.19 a
Male longevity (d)	23.53 ± 0.25 a	22.52 ± 0.31 b	20.12 ± 0.17 c	18.88 ± 0.21 d
Female longevity (d)	25.40 ± 0.27 a	24.86 ± 0.18 b	23.21 ± 0.46 c	21.19 ± 0.26 d
APOP (d)	6.20 ± 0.47 b	7.00 ± 1.36 a	7.00 ± 0.59 a	7.10 ± 0.82 a
TPOP (d)	23.53 ± 0.52 d	26.75 ± 1.44 c	29.79 ± 1.24 b	30.45 ± 1.70 a
Eclosion rate %	85.98 ± 0.13 a	80.54 ± 0.76 b	71.02 ± 0.54 c	60.66 ± 0.73 d
Fecundity (eggs laid/female)	55.59 ± 8.72 a	48.79 ± 7.08 b	32.71 ± 3.23 c	13.40 ± 3.06 d

Note: APOP, adult pre-oviposition period; TPOP, total pre-oviposition period. Data are presented as means ± SE. Different letters within a row indicate significant differences based on paired bootstrap tests (*p* < 0.05). Standard errors are estimated from 100,000 bootstrap resamples.

**Table 4:** Population parameters of F_1_ *H. vigintioctomaculata* descended from the F_0_ generation exposed to lambda-cyhalothrin.

Parameters	Concentration Treatments			
	Control	Lambda-Cyhalothrin (LC_10_)	Lambda-Cyhalothrin (LC_20_)	Lambda-Cyhalothrin (LC_40_)
	Mean ± SE	Mean ± SE	Mean ± SE	Mean ± SE
Intrinsic rate of increase, *r* d^−1^	0.087 ± 0.006 a	0.062 ± 0.007 b	0.041 ± 0.005 c	0.017 ± 0.007 d
Finite rate of increase, λ d^−1^	1.081 ± 0.006 a	1.064 ± 0.007 b	1.041 ± 0.005 c	1.017 ± 0.009 d
Net reproductive rate, *R*_0_ (offspring/individual)	25.98 ± 6.75 a	13.66 ± 3.64 b	9.16 ± 1.25 c	3.68 ± 0.96 d
Mean generation time, *T* (d)	38.97 ± 0.46 c	41.69 ± 0.74 b	53.98 ± 1.80 a	54.52 ± 1.91 a

Note: Different letters within a row indicate significant differences (*p* < 0.05).

## Data Availability

The data presented in this study are available on request from the corresponding author.
